# Dynamic Transcriptional Regulation of the Hypothalamic–Pituitary–Testis Axis in the Seasonally Breeding Teleost *Sebastes schlegelii*

**DOI:** 10.3390/ijms26052048

**Published:** 2025-02-26

**Authors:** Xueying Wang, Qinghua Liu, Jun Li

**Affiliations:** 1 CAS and Shandong Province Key Laboratory of Experimental Marine Biology, Institute of Oceanology, Chinese Academy of Sciences, Qingdao 266000, China; xueyingwang@qdio.ac.cn; 2 Laboratory for Marine Biology and Biotechnology, Qingdao Marine Science and Technology Center, Qingdao 266237, China; 3 Key Laboratory of Breeding Biotechnology and Sustainable Aquaculture, Chinese Academy of Sciences, Qingdao 266000, China

**Keywords:** *Sebastes schlegelii*, spermatogenesis, hypothalamic-pituitary-testis axis

## Abstract

Spermatogenesis, the process of male germ cell development, is tightly regulated by the hypothalamic–pituitary–testis (HPT) axis in seasonally breeding teleosts. Despite its importance, our understanding of how the brain and male germ cells coordinate key transitions—such as testis initiation and maturation—remains limited, particularly in species with distinct seasonal reproductive cycles. Black rockfish (*Sebastes schlegelii*), a marine viviparous teleost, exhibits a prolonged testis quiescent phase lasting three-quarters of the year, with testis initiation occurring in September and maturation concluding in November and December. The mechanisms underlying these transitions are poorly characterized, leaving a critical gap in our knowledge of seasonal spermatogenesis and its regulation. Addressing this gap is crucial for advancing artificial breeding technologies, which could significantly benefit the aquaculture industry. RNA-seq was used to explore the gene regulatory networks involved in testis initiation in *S. schlegelii*. Transcriptomic analyses of brain and testis were conducted across key developmental phases. In the brain, upregulated genes were notably involved in neuroactive ligand–receptor interactions, whereas in the testis, differentially expressed genes were linked to cell cycle processes and ATP-dependent chromatin remodeling. Our findings reveal the molecular mechanisms underlying testis initiation in *S. schlegelii*, providing omics evidence for the role of the HPT axis in regulating this process. By elucidating the gene regulatory networks of the brain and testis during critical transitions, this study advances our understanding of spermatogenesis in seasonally breeding teleosts. These insights pave the way for developing year-round artificial breeding technologies, contributing to the sustainable management of commercially valuable fish species.

## 1. Introduction

Spermatogenesis is a complex biological process that includes spermatogonial stem cell self-renewal, differentiation, mitosis, meiosis, and sperm maturation [1]. These processes are fundamental to male fertility across species, including fish, where significant advances have been made in understanding the molecular mechanisms underlying spermatogenesis. Research has pinpointed essential genes and pathways linked to spermatogonial stem cell markers, the initiation of meiosis, sperm maturation, and endocrine regulation [2,3,4].

In seasonally breeding species, the testis undergoes substantial structural and functional changes across different developmental phases, including variations in color, weight, and cellular composition [5,6,7,8]. The quiescence, initiation, and maturation phases are pivotal, representing transitions from spermatogonial stem cell quiescence to activation, proliferation, differentiation, and sperm maturation. The hypothalamic–pituitary–testis (HPT) axis is central to regulating these transitions in multiple species [9,10,11]. Despite this, our understanding of the mechanisms driving these developmental transitions, particularly during the initiation phase, remains incomplete.

This gap is notably apparent in* Sebastes schlegelii* Hilgendorf, a viviparous species distributed across China, Japan, and Korea. The reproductive cycle of male *S. schlegelii* encompasses five distinct phases: quiescence, initiation, development, maturation, and regression [7]. Although prior studies have examined cellular characteristics, hormone physiology, and spermatogenesis, the critical transition from quiescence to development has received limited attention. This transition represents a key regulatory point with implications for reproductive success, yet its molecular underpinnings and the role of the HPT axis remain poorly understood.

To address this knowledge gap, we investigated the transitions between the quiescence, initiation, and maturation phases of testis development in *S. schlegelii*. By integrating brain and testis perspectives, we identified highlighted crucial genes and pathways, and detailed the dynamic processes governing these transitions. Our research offers novel insights into the molecular and endocrine processes involved in testis development, enhancing the understanding of reproductive biology in seasonal breeding teleosts.

## 2. Results

### 2.1. Sequence and Assembly Analysis

Total RNA from the brain and testis was extracted using the Trizol reagent kit (Invitrogen, Carlsbad, CA, USA), followed by standard quality control procedures. Nine cDNA libraries were constructed from brain RNA and another nine from testis RNA, utilizing three biological replicates for each developmental phase. The brain libraries yielded around 63.43 Gb of sequencing data, featuring an error_rate of 0.01%, and Q20, Q30, and GC content percentages of 98.91%, 96.91%, and 47.73%, respectively. For the testis libraries, around 62.6 Gb of data was produced, featuring an error_rate of 0.027%, and Q20, Q30, and GC content values of 97.08%, 92.46%, and 50.23%.

### 2.2. Analysis of Differentially Expressed Genes

**Figure 1:**
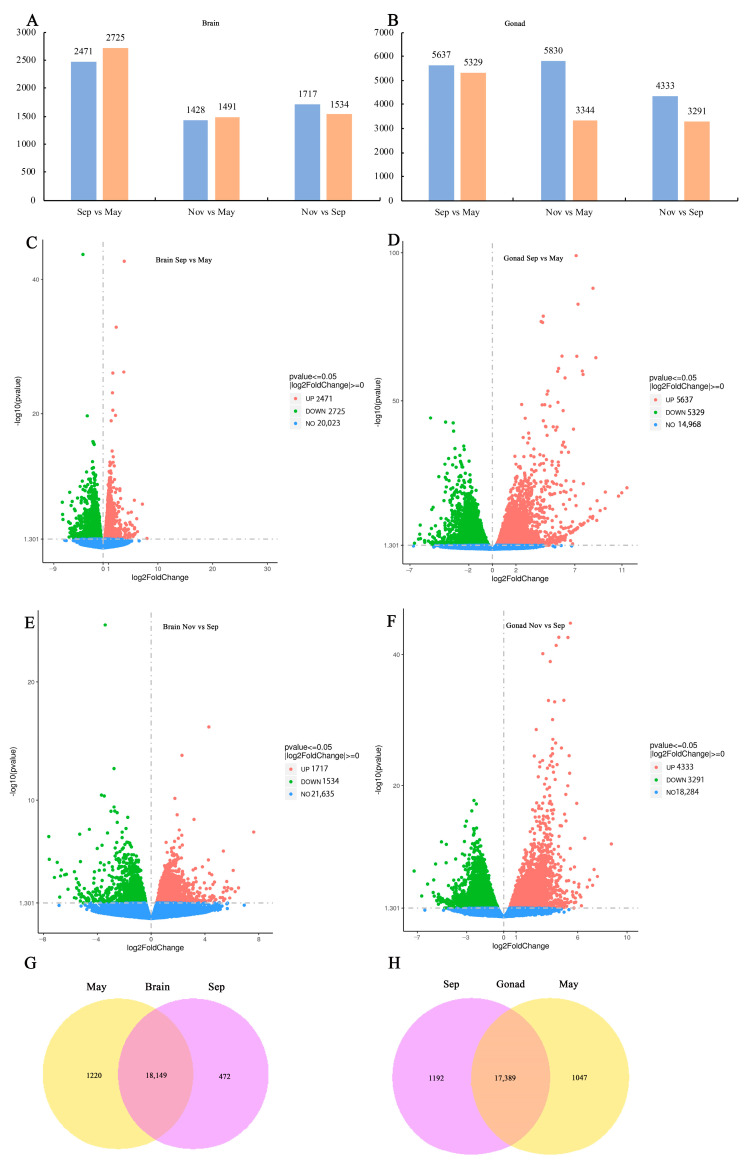
Analysis of differential gene expression across various phases. (**A**) Differentially expressed genes (DEGs) between phases in the brain; (**B**) DEGs between phases in the gonad; (**C**) volcano plots of DEGs comparing September and May in the brain; (**D**) volcano plots of DEGs comparing September and May in the gonad; (**E**) volcano plots of DEGs comparing November and September in the brain; (**F**) volcano plots of DEGs comparing November and September in the gonad; (**G**) Venn diagram of gene expression number in May and September in the brain; (**H**) Venn diagram of gene expression number in May and September in the gonad.

The differentially expressed genes (DEGs) in the brain were analyzed at various time points, with numbers ranging from 1428 to 2725 (Figure 1A). The transition from the quiescent to initiation phases exhibited the highest number of differentially expressed genes, with 2471 genes showing upregulation and 2725 genes showing downregulation (Figure 1A,C). During the transition from initiation to maturation phases, 1717 genes exhibited upregulation, while1534 genes showed downregulation (Figure 1A,E).

The DEGs in the testis were analyzed at different time points, with numbers varying between 3291 and 5830 (Figure 1B). A total of 5637 genes were upregulated and 5329 genes were downregulated between the quiescent and initiation phases (Figure 1B,D). During the transition from initiation to maturation phases, 4333 genes exhibited upregulation, while 3291 genes were downregulated (Figure 1B,F).

In the brain, 18,149 DEGs were common across phases, with 1220 DEGs specific to the quiescent phases and 472 DEGs specific to the initiation phases (Figure 1G). In the testis, 17,389 DEGs were common across phases, with 1047 DEGs specific to the quiescent phases and 1192 DEGs specific to the initiation phases (Figure 1H).

### 2.3. Enrichment Analysis of DEGs Using KEGG and Gene Ontology

**Figure 2:**
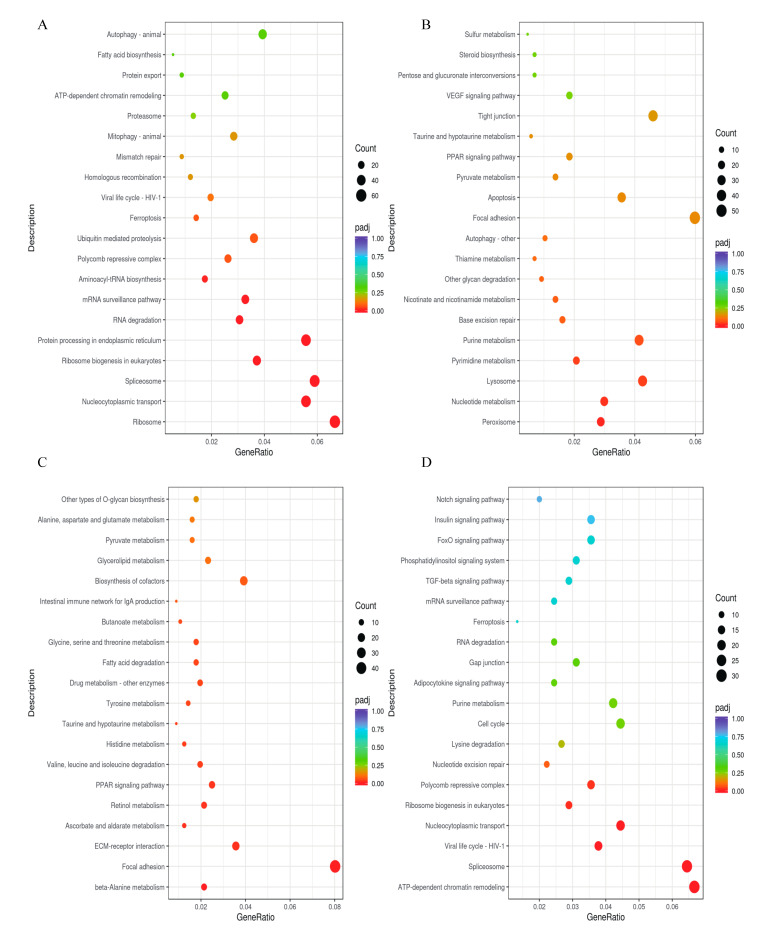

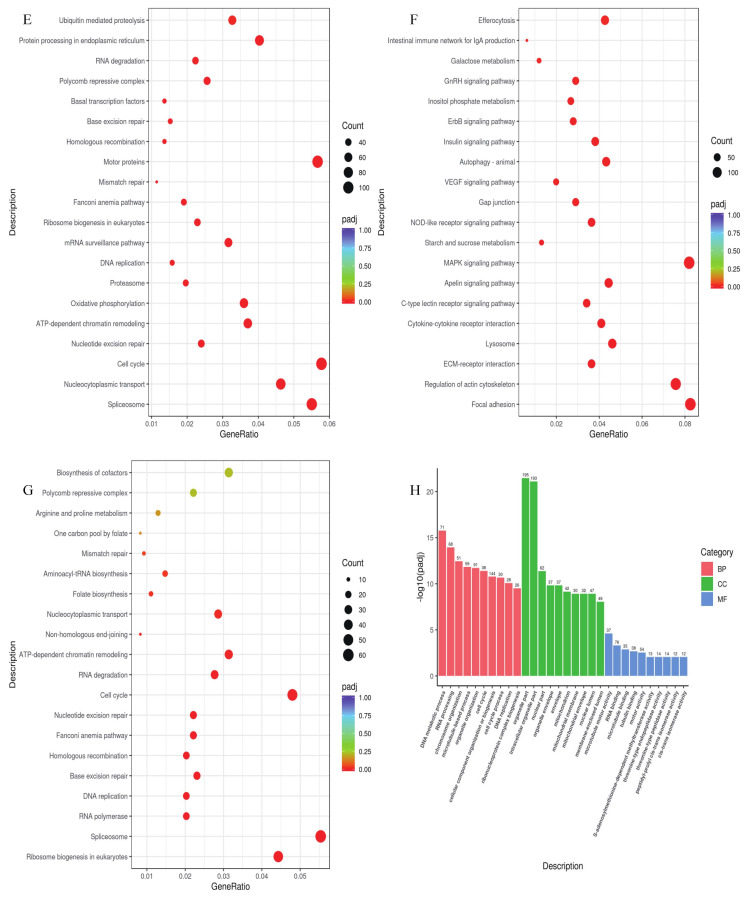
Scatter plots of enriched KEGG pathways and Gene Ontology (GO) analysis for DEGs across different phases. (**A**) Upregulated DEGs in the brain (September vs. May); (**B**) downregulated DEGs in the brain (September vs. May); (**C**) upregulated DEGs in the brain (November vs. September); (**D**) downregulated DEGs in the brain (November vs. September); (**E**) upregulated DEGs in the testis (September vs. May); (**F**) downregulated DEGs in the testis (September vs. May); (**G**) downregulated DEGs in the testis (November vs. September); (**H**) GO analysis of downregulated DEGs in the testis (November vs. September).

In the brain, upregulated DEGs during the initiation versus quiescent phases were significantly enriched in pathways related to Aminoacyl-tRNA biosynthesis, mRNA surveillance, RNA degradation, and Ribosome biogenesis in eukaryote signaling (Figure 2A). During this phase comparison, the downregulated DEGs were significantly enriched in Pyrimidine metabolism, Nucleotide metabolism, and Peroxisome pathways (Figure 2B).

In the brain, the upregulated DEGs were notably concentrated in ECM–receptor interaction, focal adhesion, retinol metabolism, and PPAR signaling pathways during the maturation compared to initiation phases (Figure 2C). In contrast, the downregulated DEGs were enriched in the TGF-beta, Polycomb repressive complex, ATP-dependent chromatin remodeling, and spliceosome pathways during this phase comparison (Figure 2D).

In the testis, upregulated DEGs during the initiation phase compared to quiescent phase were enriched in KEGG pathways related to the cell cycle, ATP-dependent chromatin remodeling, and mismatch repair (Figure 2E). The downregulated DEGs showed enrichment in the MAPK, VEGF, and insulin signaling pathways during the same phase comparison (Figure 2F). In the testis maturation phase compared to the initiation phase, downregulated DEGs were notably enriched in pathways related to mismatch repair, ATP-dependent chromatin remodeling, cell cycle, and homologous recombination (Figure 2G). The GO analysis of the downregulated DEGs in the testis during the maturation versus initiation phase revealed significant involvement in DNA, RNA processing, chromosome organization, cell cycle, DNA replication etc. (Figure 2H).

### 2.4. Gene Set Enrichment Analysis

**Figure 3:**
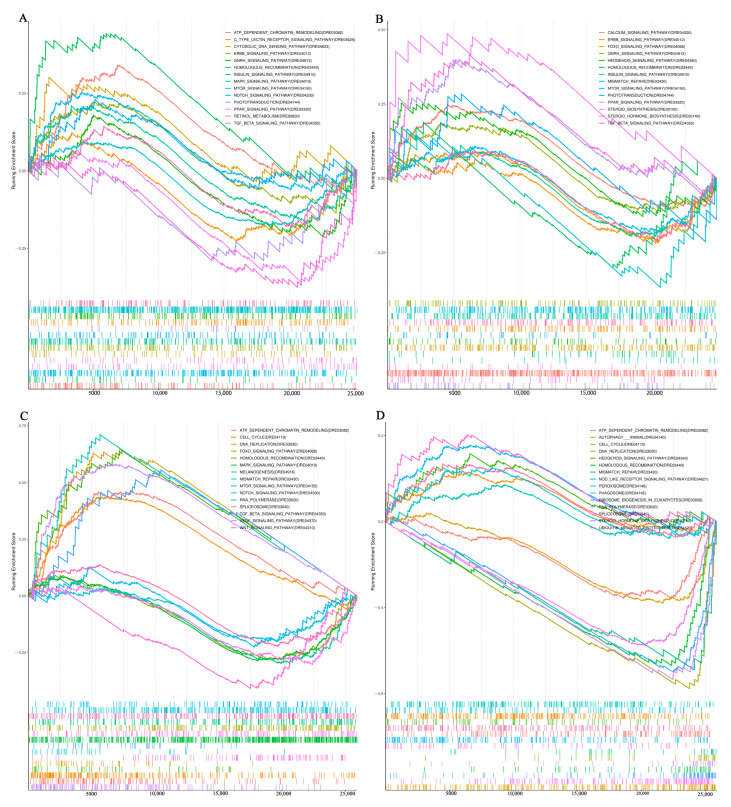
Gene set enrichment analysis. (**A**) KEGG enrichment results for September vs. May in the brain; (**B**) KEGG enrichment results for November vs. September in the brain; (**C**) KEGG enrichment results for September vs. May in the gonad; (**D**) KEGG enrichment results for November vs. September in the gonad.

The GSEA KEGG analysis of the brain indicated that the enriched pathways were linked to neuroactive processes and related functions. In the comparison between the initiation and quiescent phase, most genes were enriched in pathways including ATP-dependent chromatin remodeling, homologous recombination, mTOR, Insulin, Notch, cytosolic DNA sensing, Erbb, PPAR, TGF-beta, GnRH, MAPK, C-type lectin receptor signaling, phototransduction, neuroactive ligand–receptor interaction, and retinol metabolism (Figure 3A). In the comparison between the initiation and maturation phases, the majority of genes were enriched in pathways associated with PPAR signaling, steroid biosynthesis, Hedgehog signaling, neuroactive ligand–receptor interaction, steroid hormone biosynthesis, calcium signaling, GnRH signaling, phototransduction (Figure 3B). These enriched KEGG pathways provided a preliminary prediction of the brain involving regulating testis initiation.

GSEA KEGG analysis of the testis indicated that the enriched pathways were linked to male germ cell proliferation and meiosis. In the comparison between initiation and quiescent phases, the majority of genes were enriched in pathways including FOXO, MAPK, TGF-beta, WNT, mTOR, NOTCH, VEGF, melanogenesis, homologous recombination, mismatch repair, spliceosome, RNA polymerase, cell cycle, ATP-dependent chromatin remodeling, and DNA replication (Figure 3C). In the comparison between the initiation and maturation phases, the majority of genes were enriched in pathways associated with steroid hormone biosynthesis, ubiquitin-mediated proteolysis, phagosome, animal autophagy, peroxisome, insulin signaling, and hedgehog signaling (Figure 3D). These enriched KEGG pathways provided a preliminary prediction of the biological processes related to testis initiation.

### 2.5. DEG Validation

**Figure 4:**
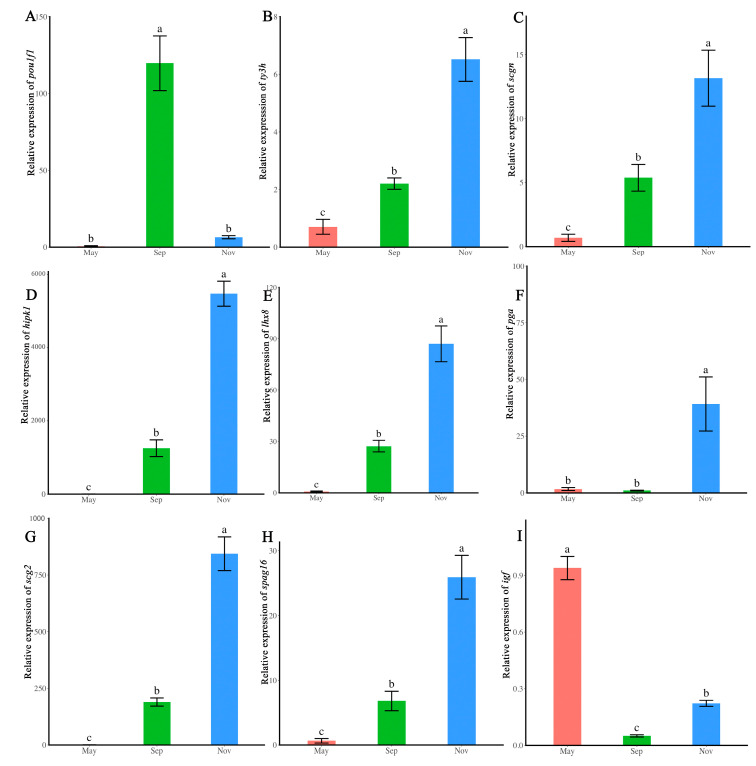
Validation of DEGs by RT-qPCR. Expression levels of *pou1f1*, *ty3h*, *scgn*, *hipk1*, *lhx8*, *pga*, *scg2*,* spag16*, and *igf* during different developmental phases. 18S was used as the reference gene. The *x*-axis represents developmental phases, while the *y*-axis depicts gene expression levels. (**A**–**C**) brain; (**D**–**I**) testis. (**A**) *pou1f1*; (**B**) *ty3h*; (**C**) *scgn*; (**D**) *hipk1*; (**E**) *lhx8*; (**F**) *pga*; (**G**) *scg2*; (**H**) *spag16*; I: *igf.* There is no significant difference between phases marked with the same letter. A significant difference exists between phases marked with different letters (*p* < 0.05).

Significant variation in gene expression levels was observed for the nine selected genes (*pou1f1*, *ty3h*, *scgn*,* hipk1*, *lhx8*,* pga*, *scg2*, *spag16*, and *igf*) across different reproductive phases (ANOVA, *p* < 0.05). In the brain, the expression of* pou1f1* was highest during the initiation phase (Figure 4A). *ty3h* and* scgn* were significantly upregulated during the maturation phase compared to the initiation and quiescent phase (Figure 4B,C). In the testis, *hipk1*, *scg2*, *lhx8*, *spag16*, and *pga* were significantly upregulated during the maturation phase compared to the initiation and quiescent phase (Figure 4D–H). Conversely,* igf* was significantly higher during the quiescent phase than during the maturation and initiation phase (Figure 4I). The RT-qPCR results confirmed the RNA-seq analysis.

### 2.6. DEG Cluster Analysis

We selected relevant genes from the hypothalamic–pituitary–testis axis related to the initiation and maintenance of quiescence for expression pattern analysis. Numerous DEGs and enriched pathways were significantly upregulated during testis initiation.

**Figure 5:**
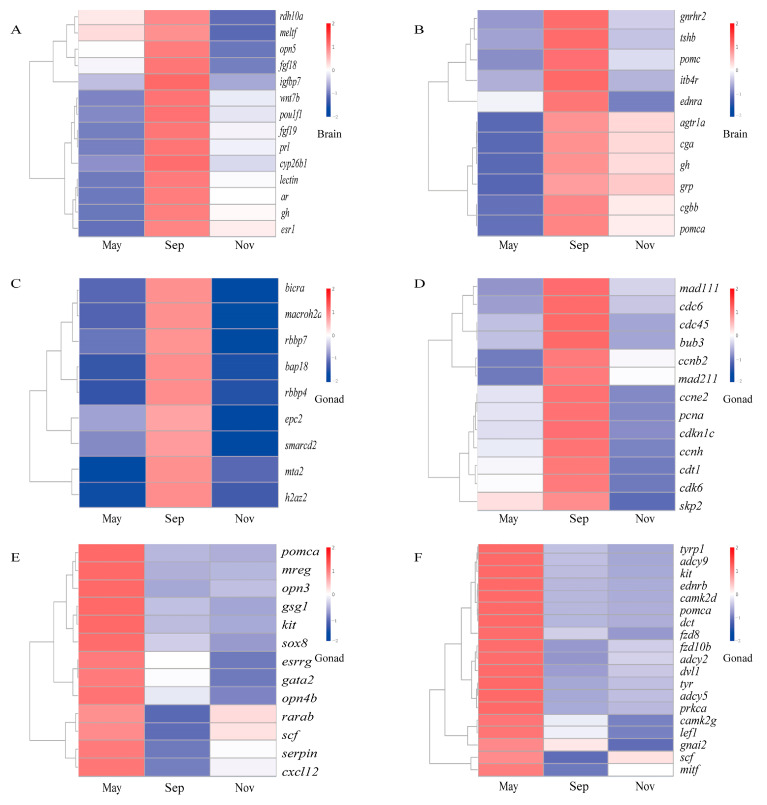
Heatmap of representative genes and significant enrichment in differential pathways. (**A**) Testis initiation-related genes; (**B**) gene enrichment in neuroactive ligand–receptor-associated pathways; (**C**) chromatin remodeling signaling pathway; (**D**) cell cycle pathway; (**E**) stemness maintenance-related genes; (**F**) melanogenesis and stemness maintenance related genes. Red and blue colors represent upregulated and downregulated transcripts, respectively.

From the brain, genes such as *rdh10a*, *meltf*, *opn5*, *fgf18*, *igfbp7*, *wnt7b*, *pou1f1*, *fgf19*, *prl*, *cyp26b1*, *lectin*, *ar*, *gh*, *esr1* (Figure 5A), and the neuroactive ligand–receptor pathway (Figure 5B) were significantly upregulated in September, corresponding to the testis initiation phase.

From the testis, genes involved in chromatin remodeling (*bicra*, *macroh2a2*, *rbbp7*, *bap18*, *rbbp4*, *epc2*, *smarcd2*, *mta2*, *h2az2*) (Figure 5C) and cell cycle pathways (*mad1l1*, *cdc6*, *cdc45*, *bub3*, *ccnb2*, *mad2l1*, *ccne2*, *pcna*, *cdkn1c*,* ccnh*, *cdt1*, *cdk6*, *skp2*) (Figure 5D) were also significantly upregulated in September during the testis initiation phase.

In contrast, for quiescence, stemness maintenance genes such as *pomca*, *mreg*, *opn3*, *gsg1*, *kit*, *sox8*, *esrrg*, *gata2*, *opn4b*, *rarab*, *scf*, *serpin*, *cxcl12* were significantly upregulated in May in testis (Figure 5E). Interestingly, genes in the melanogenesis pathway (*tyrp1*, *adcy9*, *kit*, *ednrb*, c*amk2d*, *pomca*, *dct*, *fzd8*, *fzd10b*, *adcy2*, *dvl1*,* tyr*, *adcy5*, *prkca*, *camk2g*, *lef1*, *gnai2*, *scf*, *mitf*) were also significantly upregulated in May (Figure 5F), a period when the testis remains quiescent and spermatogonia stem cells are the predominant cell type.

### 2.7. Analysis of Weighted Gene Co-Expression Network

A weighted gene co-expression network analysis (WGCNA) with scale-free topology was utilized to systematically identify co-expressed gene modules across various developmental phases. Hierarchical clustering based on topological overlap was used to identify gene groups with similar expression patterns within each module. To construct a more accurate network, low-quality genes with unstable effects on the results were excluded.

**Figure 6:**
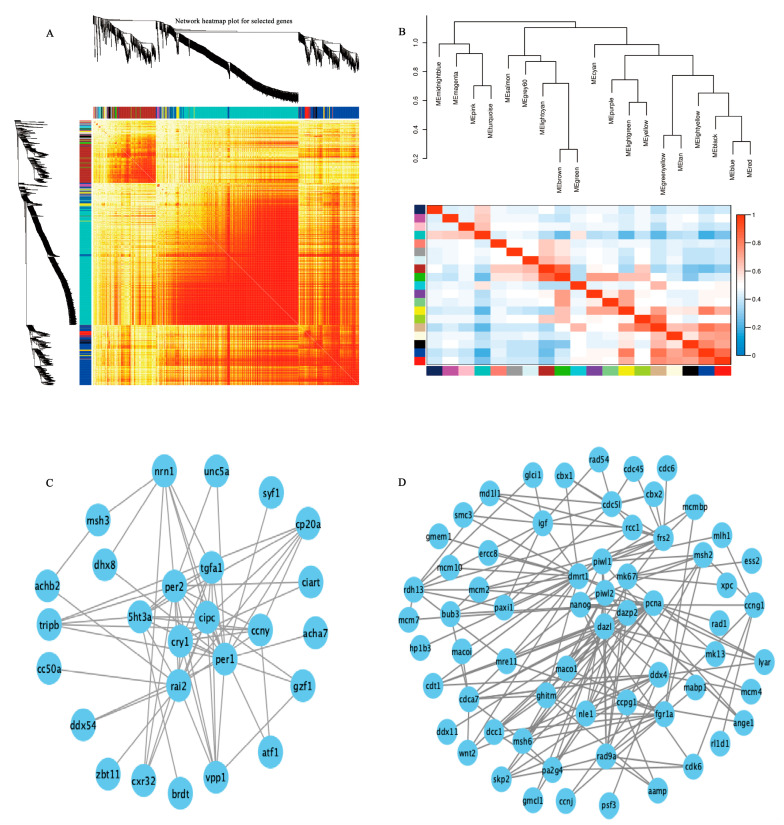
WGCNA analysis and subsequent network visualization. (**A**) Heatmap, each row and column representing a gene. Darker colors signify stronger gene connectivity. (**B**) Dendrogram illustrating module membership based on epigengenes. (**C**) Key circadian clock genes identified in the brain’s dark red module. (**D**) Key genes associated with spermatogonial stem cell proliferation and differentiation within the brown module of the gonad. Gene connectivity within each module was ranked based on connectivity values, and highly connected genes were selected based on a threshold determined by gene numbers.

We developed a hierarchical clustering tree (Figure 6A) and analyzed the power value curve to segment the modules (Figure 6B). The analysis identified 33 distinct modules in the brain based on gene expression values. Hub genes in the dark red module, including *per*, *per2*, *ccny*, *cry1*,* cipc*, *rai2*, *5ht3a*, and *tgfa1*, were significantly associated with biological clock regulation (Figure 6C). Twenty modules were identified based on testis expression values. Additionally, WGCNA identified genes with high correlation within the modules. Genes like *dmrt1*, *nanog*, *dazl*, *piwi1*, *mki67*, and *pcna*, associated with spermatogonial stem cell proliferation and differentiation, were notably enriched in the brown module (Figure 6D).

## 3. Discussion

The hypothalamic–pituitary–testis (HPT) axis plays crucial roles in synchronizing environmental cues with reproductive physiology, orchestrating seasonal reproductive cycles in male species, to adapt their breeding patterns to environmental cues. In species such as large yellow croaker (*Larimichthys crocea*) [12], European sea bass (*Dicentrarchus labrax*) [13,14], shark (*Mustelus schmitti*) [15], the HPT axis was reported to be important in coordinating testis development. In the viviparous marine species *S. schlegelii*, the initiation of testis development shifts from a quiescent to a developmental and maturation phase in response to environmental factors, particularly photoperiod and temperature. However, the molecular regulation underlying the HPT axis remains underexplored. In the present study, seasonal transcriptional variations in brain activity and changes in germ cells were investigated.

The neuroactive ligand–receptor interaction pathway was significantly upregulated and enriched during the testis initiation phase in the brain of *S. schlegelii*. In teleosts, this pathway has also been shown to play an important role in testicular activity in Nile tilapia (*Oreocchromis niloticus*) [16], yellow perch (*Perca flavescens*) [17], large yellow croaker (*Larimichthys rocea*) [18], and spotted knifejaw (*Oplegnathus punctatus*) [19]. Notably, the genes *gnrhr2*, *gh*, *tsh* were significantly upregulated in the brain during September, suggesting their potential involvement in seasonal testis initiation through neuroendocrine regulation. In this study, clock genes (*per1*, *per2*, *cry1*) were identified as hub genes in the WGCNA analysis, indicating that spermatogenetic rhythms are influenced by the circadian clock system. Previous studies have demonstrated that the expression of clock genes in the brain acts as a “timekeeper” for seasonal reproduction, while melatonin serves as an input to central circadian oscillators [20,21,22]. The expression of clock genes, along with hormones such as GnRH, NPY, and pituitary hormones like FSH, LH, GH, and PRL, regulate reproductive rhythms [23,24]. Furthermore, mutations in* cry1* or both *per1* and *per2* have been shown to affect male fertility in mice, further highlighting the circadian clock system’s critical role in spermatogenesis [25].

Studies on spermatogonial stem cell dynamics and testis development have made significant progress in recent years. The testis exhibits dynamic changes in cell characteristics across various developmental phases. The proliferative activation and differentiation of spermatogonia vary throughout different reproductive cycles in seasonally breeding animals [26,27,28,29,30], as observed in species such as rainbow trout [28] and Japanese field mice [29]. In the present study, the cell cycle pathway and chromatin remodeling signaling pathways were significantly upregulated and enriched during active reproductive periods, transitioning from quiescence to development and maturation phases. The upregulation of cell cycle gene expression indicates heightened spermatogonial stem cell proliferation and differentiation, with increased mitosis and meiosis activity. These results are consistent with findings in seasonally breeding teleosts [5,30]. Furthermore, testis pigmentation, influenced by melanogenesis, appears to play a protective role for germ cells, particularly during quiescent phases, by mitigating oxidative stress [31,32]. These findings underscore the intricate interplay between cellular processes and environmental regulation in seasonally breeding teleosts.

## 4. Materials and Methods

### 4.1. Materials

The ethical committee of the Institute of Oceanology, Chinese Academy of Sciences approved this study. Male *Sebastes schlegelii* specimens were obtained from Nanshan market, Qingdao and they were collected from cage culturing (Yantai, China). The fish were anesthetized with MS-222 (100 μg/mL). Nine testis samples were collected in May, September, and November, and nine brain samples were collected at the same time points as the testes. Three specimens were used in each month of analysis. After the sample was obtained, it was quickly put into liquid nitrogen for long-term preservation until use.

### 4.2. cDNA Library Construction and Sequencing

Separate cDNA libraries were constructed for the testes and brain at each selected time point. RNA extraction followed standard protocols, with quality assessed via 1% agarose gel electrophoresis, a NanoPhotometer^®^ spectrophotometer (IMPLEN, München, Germany), and the Qubit^®^ RNA Assay Kit (Life Technologies, Carlsbad, CA, USA) on a Qubit^®^ 2.0 Fluorometer (Life Technologies, Carlsbad, CA, USA). The Illumina NovaSeq 6000 platform (Illumina, San Diego, CA, USA) was used for sequencing. Raw data were filtered based on specific criteria to obtain high-quality clean reads.

### 4.3. Expression and Enrichment Analysis

The read count for each gene, including newly predicted ones, was determined by aligning gene positions on the reference genome and counting the reads. Reads with a mapping quality score under 10, unpaired reads, and those mapped to multiple genome regions were excluded. The featureCounts (version 1.5.0-p3) tool was used for this analysis [33].

After quantifying gene expression, statistical analysis is conducted to identify genes with significantly different expression levels across various phases. The differential expression analysis is conducted in three main steps. Initially, the read count data are normalized to adjust for sequencing depth. Next, a statistical model is applied to calculate *p*-value. Finally, adjustments for multiple hypothesis testing are made to calculate false discovery rate (FDR) values, typically represented as padj [34].

We chose suitable software to analyze gene expression differences based on the experimental conditions. Differential expression analysis utilized DESeq2 (version 1.20.0) [35], applying criteria for |log2(FoldChange)| ≥ 1 and padj ≤ 0.05 for identifying differential genes.

A heat map illustrating the DEGs across the quiescent phase (May), developmental initiation (Sep), and maturation phase (Nov) was generated based on gene expression values from the testis and brain transcriptomes. The clusterProfiler package (version 3.8.1) was utilized to perform KEGG analysis on the DEGs. Gene Set Enrichment Analysis (GSEA) was conducted using gsea v4.3.2.

### 4.4. WGCNA Analysis

Weighted gene coexpression network analysis (WGCNA) is suitable for complex data models based on gene coexpression. In the present study, all the data were used for the WGCNA analysis for testis and brain, respectively, with the WGCNA (version 1.69).

### 4.5. Validation of DEGs Using Real-Time PCR

Quantitative real-time PCR (RT-qPCR) was employed to validate the DEG data, selecting three genes (*pou1f1*, *ty3h,scgn*) from the brain library and six genes (*hipk1*,* lhx8*,* pga*, *scg2*, *spag16*, *igf*) from the testis library. Reverse transcription (AG11728) and fluorescence quantitative reagents (AG11728) were purchased from Accurate Biology, China. The reference gene was 18S. The 2^−ΔΔCt^ method was used to quantify gene expression value, with each reaction conducted in triplicate. The primers used in the study were listed in Table S1.

### 4.6. Statistical Analysis

Statistical analyses were performed using the R software (version 4.2.0). Data were presented as means ± SD. Statistical significance (*p* < 0.05) was evaluated using one-way ANOVA and Duncan’s multiple range test in R.

## 5. Conclusions

The neuroactive ligand–receptor interaction pathway and clock genes in the brain were found to be crucial regulators of testis initiation in seasonally breeding teleosts. In the testis, the activation of spermatogonia stem cells, along with processes such as cell cycle regulation and chromatin remodeling, emerged as playing critical roles during this phase transition. These findings offer important insights into the role of the hypothalamic–pituitary–testis axis and its role in coordinating reproductive cycles. Furthermore, this knowledge lays the foundation for the development of year-round artificial breeding technologies, with significant implications for aquaculture and conservation efforts.

## Data Availability

The NCBI SRA ID assigned is PRJNA1204197 and PRJNA1205967 of the sequence data for the present study.

## Supplementary Materials

The following supporting information can be downloaded at: https://www.mdpi.com/article/10.3390/ijms26052048/s1.
