# Supplementary material for: Dynamic Transcriptional Regulation of the Hypothalamic–Pituitary–Testis Axis in the Seasonally Breeding Teleost Sebastes schlegelii
Source: Int J Mol Sci. 2025 Feb 26;26(5):2048. doi: 10.3390/ijms26052048 (PMC11899799; doi:10.3390/ijms26052048)
Supplement: Supplementary file 1 [file ijms-26-02048-s001.zip › ijms-3443218-supplementary.pdf]

Supplementary Table S1. The primers used in the study.

Gene	Primer sequences	Amplification size	Tm	Amplification efficiency
pou1f1	F: TCTGTGCAGGGAAGCAAGAC<br>R: GGGAAACTCTGCAGGGTCAG	146	58	98.5%
ty3h	F: TTGGCACATGGAGGGAAGTC<br>R: GGGAATGTTGTCCGGACTGT	125	58	100.8%
scgn	F: GAGGCGATAACGGAGGACAA<br>R: GTTCTCATCCTCTGGCAGCA	126	58	99.1%
hipk1	F: TGCCTTTGGTGCATCAGTCA<br>R: CTTTACCGGAGCAGCCTTCA	119	58	98.1%
lhx8	F: ATATTTCCCCAGACGCACCC<br>R: GGCAGTTGTGTCAGCGAATG	109	58	98.3%
pga	F: ACCTGGATCCCTCTGTCCTC<br>R: CGTACTGGTTGGTTGAGGCT	190	58	98.5%
scg2	F: TCAGTTCCTCTGGCGTTCAC<br>R: GCAGGCTCTCGATGTACTCC	138	58	99.2%
spag16	F: ACTGAGTGGACGGAGATGGT<br>R: TTCTCGCTGCACGTTTCTCA	120	58	105.6%
igf	F: TGTCTGCCGCTAAGTGTCTG<br>R: GGCCAGACGTATCAGCTCTC	113	58	105.7%
18S	F: CCTGAGAAACGGCTACCACAT<br>R: CCAATTACAGGGCCTCGAAAG	119	58	97.6%
